# Role of Acetic Acid Bacteria in Food and Beverages

**DOI:** 10.17113/ftb.61.01.23.7811

**Published:** 2023-03

**Authors:** Natália Norika Yassunaka Hata, Monica Surek, Daniele Sartori, Rodrigo Vassoler Serrato, Wilma Aparecida Spinosa

**Affiliations:** 1 Departamento de Ciência e Tecnologia de Alimentos/Centro de Ciências Agrárias/Universidade Estadual de Londrina - UEL, Rodovia Celso Garcia Cid, km 380, 86057-970, Londrina, PR, Brazil; 2 Departamento de Análises Clínicas/Universidade Federal do Paraná – UFPR, Campus III – Sede Botânico, 80210-170, Curitiba, PR, Brazil; 3 Departamento de Bioquímica e Biotecnologia/Centro de Ciências Exatas/Universidade Estadual de Londrina - UEL, Rodovia Celso Garcia Cid, km 380, 86057-970, Londrina, PR, Brazil; 4 Departamento de Bioquímica e Biologia Molecular/Universidade Federal do Paraná – UFPR, Centro Politécnico, 81531-990, Curitiba, PR, Brazil

**Keywords:** acetic acid, food, beverage, oxidative fermentation

## Abstract

Acetic acid bacteria (AAB) are microorganisms widely distributed in nature. Although this group is involved in the spoilage of some foods, AAB are of great industrial interest, and their functionality is still poorly understood. AAB convert ethanol, sugars and polyols into various organic acids, aldehydes and ketones *via* oxidative fermentation. These metabolites are produced during a succession of biochemical reactions in various fermented foods and beverages, such as vinegar, kombucha, water kefir, lambic and cocoa. Furthermore, important products such as gluconic acid and ascorbic acid precursors can be produced industrially from their metabolism. The development of new AAB-fermented fruit drinks with healthy and functional properties is an interesting niche for research and the food industry to explore, as it can meet the needs of a wide range of consumers. Exopolysaccharides such as levan and bacterial cellulose have unique properties, but they need to be produced on a larger scale to expand their applications in this area. This work emphasizes the importance and applications of AAB during the fermentation of various foods, their role in the development of new beverages as well as numerous applications of levan and bacterial cellulose.

## INTRODUCTION

Acetic acid bacteria (AAB) are mesophilic, Gram-negative bacteria that belong to the *Acetobacteraceae* family. They can be single, in pairs or chains and have an ellipsoidal to elongated shape (rods). Their width varies from 0.4 to 1.0 µm, and their length ranges from 0.8 to 4.5 µm. AAB do not sporulate (*1*).

They show positive catalase and negative oxidase reactions, and have a strictly aerobic metabolism with oxygen as the terminal electron acceptor (*1*). According to Laureys *et al.* (*2*), AAB grow well between pH=5.0 and 6.5, but can also grow at pH=3.0-4.0 and even lower. The optimum temperature for growth is between 25 and 30 °C, and typically, no growth occurs above 34 °C (*2*, *3*). However, thermotolerant strains can continue to grow at 37 °C, and some strains can even grow at temperatures as high as 42 °C (*4*, *5*).

To date, twenty genera have been described in the family *Acetobacteraceae*, among which the ones with the highest number of species are *Acetobacter, Gluconobacter, Asaia, Komagataeibacter* and *Gluconacetobacter* (*6*, *7*). The group can oxidize various types of sugars, sugar alcohols and alcohols to their respective aldehydes, ketones and corresponding organic acids through an incomplete oxidation process called oxidative fermentation, from which they obtain their energy (*3*). *Acetobacter* and *Komagataeibacter* spp., for example, are specialized in converting ethanol to acetic acid *via* two successive oxidative steps and are thus common in alcoholic and acidic environments, such as the vinegar industry (*8*, *9*). They also have a complete set of citric acid cycle (CAC) enzymes, which are required for further oxidation of organic acids to CO_2_ and H_2_O (*3*). In contrast, *Gluconobacter* spp. occur preferentially in sugar-rich niches and are particularly proficient in the oxidation of sugars and sugar alcohols (*10*, *11*). Due to a lack of CAC enzymes, *Gluconobacter* species are unable to oxidize acetate to CO_2_ and H_2_O, but they are useful in the biotechnological synthesis of precursor compounds of vitamin C (l-sorbose), gluconic acid, and its derivatives, dihydroxyacetone and miglitol (*12*, *13*).

The bacterium *Gluconacetobacter diazotropicus* is the most well-known member of the genus *Gluconacetobacter*, which plays an important role as nitrogen-fixing bacteria in plants. In addition, *G. diazotropicus* produces indole-3-acetic acid and gibberellins A1 and A3, which are important hormones that control plant growth (*14*, *15*).

**Fig. 1:**
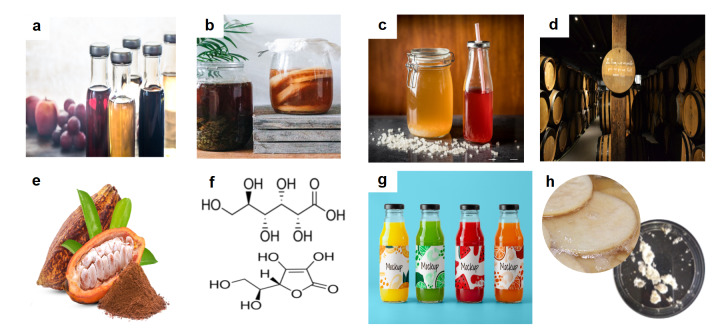
Acetic acid bacteria are involved in the production of various foods, beverages, chemicals and exopolysaccharides: a) vinegar types from different raw materials, b) kombucha, c) water kefir, d) lambic, e) cocoa, f) organic acids (gluconic and ascorbic), g) new fruit drinks, and h) exopolysaccharides (bacterial cellulose and levan). Parts of the figure designed by iStock

The genus *Asaia*, on the other hand, has been linked to beverage spoilage and has recently been found to be a symbiotic microorganism in malaria-carrying mosquitos (*16*). In addition, the role of AAB in the production of exopolysaccharides (EPS) is highlighted, the most valuable being bacterial cellulose and levan, produced mainly by the species of *Komagataeibacter*, *Kozakia*, *Gluconacetobacter*, *Neoasaia* and *Gluconobacter* (*3*, *8*). AAB are widely distributed in alcoholic, sugar-rich and acidic environments (*14*). They are often seen only as spoilage agents in wine, where acidity is undesirable, but their role in the production of fermented foods such as vinegar, kombucha, water kefir, lambic and cocoa, also in the bioconversion of specific products, such as ascorbic acid, as well as the applicability and functionality of levan and bacterial cellulose (Fig. 1) is still quite limited. Therefore, the objective of this review is to demonstrate the many advantages of AAB in this area, also encouraging their research in other application areas.

## AAB IN FOOD AND BEVERAGE FERMENTATIONS

### Vinegar

Vinegar production has been around for over 10 000 years (*3*). Despite not being considered a ’food’ and not having a high nutritional value, vinegar is consumed by people of all social classes all over the world, and it differs in terms of the used raw materials, manufacturing technologies and its wide range of applications (Fig. 1a) (*3*, *17*).

The definition and standards of vinegar identity and quality have some local differences, but in general, food regulatory agencies consider vinegar to be the result of a double fermentation (first alcoholic, then acetic) of sugar-rich substrates (*18*). In Brazil, the MAPA (Ministry of Agriculture, Livestock and Supply) defines a product as fermented by acetic acid if the minimum volatile acidity is 4% (g/100 mL, expressed in acetic acid) obtained from acetic fermentation of fruit, cereal, other vegetables, honey, vegetable mixture or hydroalcoholic mixture already fermented in alcoholic fermentation. The acetic acid fermented product can be called a *vinegar of* plus *the name of the used substrate* (*19*).

The substrate used in the processing of vinegar is mostly of plant origin, including fruits (*e.g.* grapes, apple, mango, *etc.*), cereals, onion and cider, except for honey vinegar and whey vinegar (*18*, *20*). The chemical composition of the raw material has a strong influence on the selection of microorganisms and determines the dominant species involved in the acetification process (*21*). Table 1 shows the main AAB species involved in vinegar production (*1**,**7**,**22**-**60*).

**Table 1:** Acetic acid bacteria (AAB) associated with fermented foods and beverages and with the synthesis of chemical compounds and polysaccharides

Food/Beverage /Chemical compound/Polysaccharide		AAB species associated with the fermentation									Reference
Vinegar											
Fruit vinegar (apple cider, orange, red and white wine, persimmon, white and red grape, apricot and blueberry)		*Acetobacter pasteurianus, A. aceti, A. estunensis, A. pomorum, A. syzygii, Komagataeibacter europaeus, Novacetimonas hansenii* (formerly *K. hansenii*), *K. kakiaceti, K. oboediens, K. melaceti, K. melomenusus, N. pomaceti* (formerly *K. pomaceti*), *K. rhaeticus, K. saccharivorans, Gluconacetobacter entanii, N. maltaceti* (formerly *K. maltaceti*), *K. nataicola, K. intermedius, K. xylinus* and *Gluconobacter oxydans*									*1,7,22-27*
									
Cereal vinegar (rice grain and rice hull, wheat bran and glutinous rice)		*K. europaeus, K. kakiaceti* and *K. medellinensis*									*27-29*
									
Cheese whey vinegar		*A. aceti* and *A. pasteurianus*									*30,31*
Kombucha											
				
		*A. papayae, A. indonesiensis, A. lovaniensis, A. okinawensis, A. peroxydans, A. syzgii, A. tropicalis, K. takamatsuzukensis, K. oboediens, K. eurapaeus, K. saccharivorans, K. intermedius, K. xylinus, K. rhaeticus, Novacetimonas hansenii* (formerly *K. hansenii*), *Gluconacetobacter liquefaciens, G. entanii, Gluconobacter cerinus, G. oxydans* and *Tanticharoemia sakaeratensis*									*32–37*
Water kefir														
		*A. indonesiensis, A. fabarum, A. orientalis, A. tropicalis, A. okinawensis, A. lovaniensis, A. lovaniensis, K. intermedius, K. saccharivorans, Novacetimonas hansenii* (formerly *K. hansenii*), *G. cerinus, G. japonicus* and *G. liquefaciens*									*38-40*
Lambic														
		*A. orientalis, G. cerevisiae, G. wancherniae, A. pasteurianus, A. aceti, A. lovaniensis, A. lambici* and *A. pomorum*									*41–43*
Cocoa														
		*A. pasteurianus, A. syzygii, A. tropicalis, A. ghanensis, A. indonesiensis, A. okinawensis, Novacetimonas hansenii *(formerly *K. hansenii*)*, G. oxydans, G. frateurii, G. diazotrophicus* and *Granulibacter bethesdensis*									*44-48*
Gluconic acid, dihydroxyacetone, vitamin C precursors, miglitol											
		*G. oxydans*									*8,12,49,50*
New beverages from AAB											
		*Acetobacter* sp. and *G. japonicus*									*51-53*
Levan														
		*G. albidus, G. cerinus, G, oxydans, G. frateurii, Kozakia baliensis, Neoasaia chiangmaiensis, Tanticharoenia sakaeratensis, Novacetimonas hansenii *(formerly *K. hansenii*)*, K. xylinus, A. pasteurianus* and *G. diazotrophicus*									*54-58*
Bacterial cellulose											
		*Novacetimonas hansenii* (formerly *K. hansenii*), *K. nataicola*, *K. rhaeticus*, *K. swingsii*, *K. maltaceti* and *K. xylinus*									*24,59,60*

Generally, vinegar is made through two fermentation processes: alcoholic fermentation and acetic acid fermentation (*61*). Under anaerobic conditions, yeasts (typically strains of *Saccharomyces cerevisiae*) convert fermentable sugars to ethanol, whereas in acetic acid fermentation, AAB convert ethanol to acetic acid by the activity of two membrane-bound enzymes located on the outer surface of the cytoplasmic membrane (periplasmic side) (*3*, *18*). First, alcohol dehydrogenase (ADH) oxidizes ethanol to acetaldehyde, which is then oxidized to acetic acid by aldehyde dehydrogenase (ALDH) (*61*). On the other hand, some rice and cereal vinegar made from starchy raw materials include a distinct saccharification step prior to alcoholic fermentation (*62*).

Three main methods are used in industrial vinegar production: (*i*) the slow, traditional Orleans or French (surface acetification carried out in wooden barrels), (*ii*) the fast generator (production under forced aeration with wood chips or other inert material), and (*iii*) the rapid submerged (modern or industrial; batch acetification with forced aeration and agitation) (*18*, *63*). In the production of traditional vinegar, the acetification is typically made using a culture from a previous batch known as ’seed-vinegar’ or ’mother of vinegar’, whereas selected cultures are added to ensure large-scale production, higher yield, better safety, process stability, shorter fermentation time and product losses, as well as avoiding undesirable characteristics caused by uncontrolled fermentation (*26*, *64*). The use of specific AAB starters, however, is still far from being a common practice (*26*).

The microbiota that leads to vinegar production is complex and includes several genera of AAB; however, *Acetobacter* and *Komagataeibacter* species have a strong capacity to produce acetic acid, and both genera also exhibit high resistance to high ethanol and acetic acid concentrations, which are essential characteristics required for industrial vinegar production (*3*, *65*). Thermotolerant AAB have also been introduced for the production of a variety of valuable products, including vinegar (*50*). They are advantageous for industrial vinegar fermentation because they allow for stable fermentation with lower cooling costs, particularly in tropical countries (*66*, *67*).

Vinegar has a wide range of applications. Different types of vinegar are frequently used as preservatives, flavouring agents, and as ingredients in mayonnaise, salad dressings, mustard and other condiments (*17*, *20*, *68*). Its use as a routine medicine for humans and animals dates back to remote antiquity; in addition, it can be used as a cleaning agent and in some countries even as a healthy drink (*3*, *17*).

Although vinegar is traditionally used as a flavouring and food preservative, recent scientific studies have reported that regular consumption of vinegar can promote beneficial physiological health effects (*3*, *65*, *69*). Among the therapeutic properties of vinegar are its antibacterial activity, regulation of blood pressure and glycaemia, antioxidant activity, prevention of cardiovascular diseases, and prevention of obesity (*8*, *70*). In addition to acetic acid, some types of vinegar contain several bioactive compounds, such as polyphenols, that contribute to their taste, smell, and specific functions. Considering that different types of vinegar can be produced using different raw materials, processes, and species of AAB, understanding the relationship between the compounds present and the functionality of vinegar is of great importance (*20*, *70*).

### Kombucha

Kombucha is a popular drink usually consumed in Asia (*35*) (Fig. 1b). It is a nonalcoholic refreshing beverage, with accentuated acidity and a specific flavour (*71*). Traditionally, it is made by fermenting sweetened tea, but other plant (*e.g*. cereals or leaves) or animal (*e.g.* milk) raw materials, as well as mushrooms, can also be used (*72*). Fermentation lasts approx. 7 to 10 days and occurs quickly after adding the cellulosic layer called tea fungus or SCOBY (symbiotic colony of bacteria and yeast) to the sweetened tea (*73*). The microbial composition of kombucha varies significantly from batch to batch depending on its origin, substrate and fermentation conditions (*74*). It predominantly comprises AAB, which include *Komagataeibacter* and *Gluconobacter,* while *Acetobacter* species are less abundant (*71*). *Komagataeibacter xylinus* is considered one of the most important species involved in the fermentation of kombucha due to its superior capacity for cellulose synthesis (*37*). Table 1 shows the main AAB species involved in kombucha fermentation (*32*–*37*).

In addition to AAB, a wide range of yeast species can be found in the kombucha, including species of the genera *Zygosaccharomyces*, *Candida*, *Kloeckera/Hanseniaspora*, *Torulaspora, Pichia, Brettanomyces/Dekkera, Saccharomyces, Lachancea, Saccharomycoides, Schizosaccharomyces* and *Kluyveromyces* (*34*, *75*). Lactic acid bacteria (LAB), such as *Lactobacillus, Lactococcus* and *Bifidobacterium* species, can occur (*75*); however, they do not seem to be a crucial part of the kombucha microbial ecosystem since they are not always present (*2*).

In this symbiotic relationship, the yeast community hydrolyses the sucrose present in the medium to glucose and fructose, and produces ethanol from glucose. AAB use glucose, fructose and ethanol to produce gluconic acid, glucuronic acid, acetic acid, d-saccharic acid-1,4-lactone and bacterial cellulose (*37*, *71*). The produced acetic acid further stimulates the production of ethanol by the yeasts, and this ethanol is then converted to acetic acid by AAB. The continuous accumulation of ethanol and acetic acid in the medium prevents contamination by various pathogenic microorganisms (*73*).

The composition of kombucha also includes vitamins, phenolic compounds, amino acids and some hydrolytic enzymes (*35*, *75*). The chemical profile of kombucha may be responsible for its multiple health benefits when associated with regular consumption of the drink (*35*). Recently, tea has attracted the attention of researchers and consumers due to its *in vitro* biological activities, such as antimicrobial, antioxidant and anti-inflammatory activities, and anticarcinogenic potential. However, more clinical investigations and *in vivo* evaluations should be carried out to confirm the health benefits of the beverage (*75*, *76*).

### Water kefir

Water kefir is a sparkling, refreshing, low-alcohol drink with acidic and fruity flavours (*71*) (Fig. 1c). It is obtained by the spontaneous fermentation of sugar solution, dried fruits, and water kefir grains (insoluble dextran). These translucent grains, whose shape resembles that of a cauliflower, contain microorganisms that act as an inoculum for the fermentation process (*77*).

The microbial composition of the grains consists of yeasts, LAB and AAB, with the bacterial community presenting a higher diversity. Some of the main microorganisms of water kefir are LAB, such as *Lentilactobacillus hilgardii*, *Liquorilactobacillus nagelii*, *Lacticaseibacillus paracasei* and *Bifidobacterium aquikefiri*, and yeasts such as *Saccharomyces cerevisiae* and *Dekkera bruxellensis* (*39*, *40*). During the first 24 h of fermentation, yeasts (mainly *S. cerevisiae*) convert sucrose to alcohol. Furthermore, yeasts hydrolyze sucrose by the action of invertase, promoting an increase in glucose and fructose, which are then metabolized by LAB and AAB (*3*, *39*). In the advanced stages, ethanol concentrations decrease due to oxidation of ethanol to acetic acid by AAB (*e.g. Gluconobacter*, *Komagataeibacter* and *Acetobacter*) (*40*). Table 1 shows the main AAB species involved in water kefir fermentation (*38*–*40*).

At the end of fermentation, the main products are ethanol, lactic acid, acetic acid and other metabolites, such as mannitol, glycerol, esters, aldehydes and other organic acids (*78*, *79*). Kefir drinks, such as kombucha, have been linked to numerous health benefits. The beverage is well known for having potentially ’probiotic’ and antimicrobial properties against a wide range of pathogenic bacteria (*78*, *80*). Furthermore, studies have shown that water kefir has antihyperlipidemic properties (*81*), antioxidant (*39*, *82*) and anticarcinogenic activities, hepatoprotective, anti-inflammatory and gastroprotective effects, among others (*79*). Due to the numerous positive effects of kefir, several substrates have been investigated for the adaptation of its grains (*39*, *82*–*84*). This has enabled the emergence of new functional drinks with characteristics similar to those of the traditional brown sugar kefir (*78*).

### Lambic

Lambic, originally from Belgium, is probably one of the oldest beer styles brewed to date (*85*, *86*) (Fig. 1d). It is a refreshing, alcoholic, acidic beer with fruity notes and only a few residual carbohydrates. The spontaneous fermentation occurs in the presence of water, barley malt, unmalted wheat and aged dry hops, and maturation takes up to three years in wooden barrels (*71*).

An in-depth examination of the dominance of microbial profile of lambic fermentation during three years revealed four distinct phases (*21*). Table 1 shows the main AAB species involved in lambic fermentation (*41*–*43*). The first phase (from the start up to 1 month of fermentation) begins with the members of the *Enterobacteriaceae* family, which are inhibited by ethanol accumulation produced by wild (oxidative) yeast, acidification by enterobacteria and AAB (*A. orientalis*) and glucose reduction by microbial growth in general (*71*, *87*). *Gluconobacter* species such as *G. cerevisiae* have also been isolated during this phase, probably as a result of the combination of a monosaccharide-rich environment and low ethanol concentrations (*42*).

The second phase of ethanol fermentation, referred to as the most important, extends until the fourth month, with yeasts (*Saccharomyces cerevisiae*, *S. bayanus/pastorianus* and *S. uvarum*) being the main representatives for the conversion of carbohydrates into ethanol and carbon dioxide (*88*). After 4 to 10 months of fermentation, the acidification phase (third phase) takes place. This phase is characterized by the predominance of the LAB and AAB species, which together produce lactic and acetic acids, resulting in a pH drop below 3.5 (*41*, *87*). During this fermentation stage, the most frequently isolated LAB species are homofermentative *Pediococcus damnosus* and heterofermentative *Lactobacillus brevis*, whereas the most frequently reported AAB species are *A. pasteurianus* and *A. lambici.* LAB species produce both lactic and acetic acid from saccharides, while AAB species oxidize ethanol to acetic acid and produce acetoin from the lactic acid produced by LAB species (*41*, *89*).

After the acidification phase, the final or maturation phase begins and can last for several years (*89*). LAB, AAB and primarily *Brettanomyces* yeast species are present during this phase. The LAB and AAB species that proliferate during this phase are typically the same species that are present in the previous phase (*41*). *B. bruxellensis* and other species belonging to the genus *Brettanomyces* play a key role in the final flavour formation of lambic (*90*) since they synthesize precursor compounds responsible for the characteristic Brett flavour (volatile phenolic compounds 4-vinylguaiacol and 4-vinylphenol) of lambic and several ethyl esters, such as ethyl acetate and ethyl lactate. Together with *Brettanomyces,* AAB may also participate in ethyl acetate formation (*43*).

AAB are abundant during major periods of the first fermentation year of traditional lambic production, producing much larger concentrations of acetic acid and acetoin (from in the liquid/air interphase of the casks). The formation of acetic acid by AAB and the subsequent formation of ethyl acetate are desired compounds for complex lambic; however, excessive AAB development must be controlled to avoid an unfavourable flavour profile (*42*, *87*).

### Cocoa

Cocoa bean (*Theobroma cacao* L.) is the main raw material for the manufacture of chocolate (*91*) (Fig. 1e). Taken from freshly harvested cocoa pods, raw cocoa beans are subjected to a complex fermentation that involves physical and biochemical transformations in all bean structures and lasts 3–7 days depending on various factors including the seed genetic origin, agro-ecological conditions and the used method (*92*, *93*). This process is primarily regulated by yeast, LAB, AAB and *Bacillaceae* (particularly *Bacillus*) that use the cocoa bean pulp as a growing substrate. Several molecules are released during microbial fermentation of cocoa, giving chocolate its distinctive aromatic profile, reducing bitterness and astringency, and finally killing the embryo to prevent its germination (*94*).

During the fermentation of cocoa beans, three main stages can be identified (*94*). Yeasts are the most prevalent microorganisms in the first 24 h of fermentation, converting pulp sugars to ethanol and carbon dioxide *via* alcoholic fermentation (anaerobiosis) (*45*). They are involved in the production of pectinolytic enzymes, which degrade pectin and allow oxygen to enter the cocoa pulp (*93*). Furthermore, yeasts produce a large number of aroma compound precursors that significantly contribute to the development of the chocolate aroma profile (*47*). Alternatively, simultaneously with yeast, fructophilic LAB species use fructose as an energy source, with or without citric acid conversion, and heterofermentative LAB species grow by converting glucose to lactic acid, acetic acid, ethanol, carbon dioxide and/or mannitol (*95*).

The second stage is distinguished by an increase in the lactic acid concentrations as a result of an increase in the LAB populations and a reduction in yeasts (*94*). Lactic acid produced in seeds is important to activate endogenous enzymes and contribute to the generation of chocolate flavour and aroma (*45*).

During the third phase, the high ethanol concentrations (metabolized by yeast) and oxygen ingress because of pulp liquefaction provide conditions for the growth of AAB (*44*). Table 1 shows the main AAB species involved in cocoa bean fermentation (*44*–*48*). Ethanol, which is the main energy source, is converted into acetic acid by AAB, particularly *Acetobacter* species, while lactic acid produced by LAB serves as the primary carbon source (*95*). Lactic acid is primarily oxidized into acetoin and, to a lesser extent, acetic acid due to low pyruvate decarboxylase activity in *Acetobacter* (*95*). The entry of acetic acid and ethanol, along with the temperature increase (approx. 50 °C), causes the death of the seed embryo and induces a series of endogenous reactions that produce flavour, aroma and colour precursors of the chocolate raw material (*47*, *96*). As a result, the counts of yeast, LAB and AAB decrease, favouring the growth of *Bacillus* spores in the later stages of cocoa bean fermentation (*94*, *95*).

Among the different genera of AAB, *Acetobacter* is the most common during cocoa fermentation (*45*). It includes *A. ghanensis* and *A. senegalensis,* which predominate mainly at the beginning of fermentation, whereas *A. pasteurianus* and occasionally *Acetobacter lovaniensis, Acetobacter syzygii* or *Acetobacter tropicalis* prevail during the last phase, when the concentration of ethanol is high (*45*, *95*). *A. pasteurianus* prevails during the fermentation of cocoa due to its ability to oxidize ethanol, lactic acid and mannitol, as well as its tolerance to acidity and heat (*95*). On the other hand, *Gluconobacter* species can be prevalent at the beginning of the fermentation due to their preference for sugar metabolism. However, their growth is undesirable, as this can result in the production of gluconic acid from glucose and off-flavours, impacting the final quality of cocoa beans (*45*).

## OTHER METABOLITES PRODUCED BY AAB

AAB can participate as biocatalysts in the industrial manufacturing of a wide range of compounds, in addition to being employed commercially in the manufacturing of vinegar and other fermented foods (*8*, *14*) (Fig. 1f). *Gluconobacter* strains, in particular *G. oxydans*, can carry out oxidative fermentation of sugars and sugar alcohols, resulting in the formation of l-sorbose, ketogluconic acids, dihydroxyacetone (DHA) and cyclic ketones, among other compounds (*21*, *50*). The oxidative fermentation of l-sorbose from d-sorbitol is the most classic example observed during the production of vitamin C by *Gluconobacter*. Other precursor intermediates, such as 2-keto-d-gluconic acid (2KGA) from gluconic acid, 2,5-diketo-d-gluconic acid (25DKGA) and 5-keto-d-gluconic acid (5KGA), are also present in the synthesis route (*14*, *21*). The 5KGA has potential applications in the synthesis of tartaric and xylaric acids, in addition to being a precursor for the manufacture of aromatic compounds such as 4-hydroxy-5-methyl-2,3-dihydrofuranone-3, a valuable product used in the food industry (*97*). The microbial production of DHA from glycerol has been explored in the pharmaceutical industry, and it can be used as a tanning agent and as an intermediate for the synthesis of various chemicals and surfactants (*14*, *98*). *Gluconobacter* species can also be applied in the biotransformation of miglitol precursors, a drug used for the treatment of type II diabetes; in the production of gluconic acid, considered a multifunctional acid in the food, feed, beverage, textile, pharmaceutical and construction industries; and in the manufacture of shikimic acid, a key intermediate for numerous antibiotics (*8*, *12*, *49*, *50*) (Table 1).

## DEVELOPMENT OF NEW PRODUCTS FROM AAB

Currently, consumers have shown a growing interest in foods that, in addition to satisfying their hunger, can also prevent nutrition-related diseases and improve mental health (*99*). According to the Food and Agriculture Organization of the United Nations (FAO), fruits constitute an important part of a healthy diet. In addition to being a source of dietary fibre, vitamins, minerals and beneficial phytochemicals, fruits may help lower the risk factors for diseases such as overweight/obesity, chronic inflammation, high blood pressure and high cholesterol (*100*).

Fruit-based fermentation has improved the nutritional and functional quality of beverages. In addition, rising consumer demand for lactose-free products with low fat content and few additives makes this type of fermentation a promising tool for meeting the needs of obese people with cardiovascular diseases, allergies, intolerances, vegans and vegetarians (*101*–*103*).

The development of fruit drinks containing probiotic bacteria stands out among current research (*104*–*106*). These microorganisms improve mineral bioavailability, digestibility and organoleptic properties such as colour, flavour and aroma, in addition to providing a functional beverage (*103*). However, the acidic environment, as well as the presence of anti-nutritional and inhibitory factors in fruits, make maintaining bacterial viability and stability during processing and storage a major challenge (*102*). Given this, the fermentation of fruit drinks with AAB becomes a viable alternative, since they can oxidize a wide range of substrates and are typically found in sugar-rich and highly acidic environments (Table 1).

Fruit vinegar drinks, for instance, are gaining popularity in North America (Fig. 1g). By definition, the product must be made from at least one type of fruit and contain at least 300 g of fruit juice for each litre of fermented product. These beverages have been categorized according to the volume fraction of acetic acid as drinks with low acidity (*φ*<3%) and high acidity (*φ*=5-7%) (*107*). Furthermore, the used fermentation method and the volume fraction of acetic acid may affect the content of total sugars and soluble solids, titratable acidity and density (*107*). Regarding its benefits, *in vivo* animal studies have shown that tomato vinegar drinks can prevent visceral obesity and insulin resistance (*51*), while pomegranate vinegar drinks have been shown to reduce visceral adipose tissue in humans (*108*). Other research suggests that fruit vinegar drinks such as cranberry, blueberry and tomato could be used to treat hypertension and hypercholesterolaemia (*107*). However, according to Chang *et al.* (*109*), continuous consumption of vinegar drinks should be avoided to prevent gastrointestinal injuries.

Another recent approach has been focused on gluconic acid fermentation. Although works related to this type of fermentation are still scarce, their results are very promising. The gluconic acid fermentation of strawberry drink by *G. japonicus* converts glucose into gluconic acid while keeping the fructose naturally present in the fruit as a sweetener. This allows diabetics to consume a drink that keeps phenolic compounds (nonanthocyanins) and antioxidant activity practically unchanged (*110*). Furthermore, its composition remains stable for 15 days at room temperature (27−30 °C) and up to 30 days when refrigerated (4 °C) (*53*). In another study, Hornedo-Ortega *et al*. (*111*) compared the antioxidant activity and anthocyanin composition of alcoholic and gluconic acid-fermented strawberry drinks. The authors concluded that the gluconic acid fermentation of strawberry beverages by *G. japonicus* is a novel process that preserves the anthocyanin composition and shows higher antioxidant activity values than alcoholic fermentation (*111*). Ordóñez *et al.* (*112*) also confirmed the safety of strawberry drink fermented with gluconic acidby demonstrating that none of the eight studied biogenic amines was detected. In addition to the bioactive compounds found in fruits, gluconic acid and its derivatives have been shown to have prebiotic properties. This acid promotes the growth of *Lactobacillus* sp. and *Bifidobacterium adolescentis* in the human colon and alters the metabolic profile of the intestines (*113*). Gluconic acid and its derivatives are approved for use in food and are commonly used to preserve and/or improve the sensory properties of dairy products and soft drinks (*113*). The presence of higher proportions of gluconic acid in kombucha, for example, improved the taste of the drink in a study conducted by Li *et al.* (*114*). Gluconic acid contributes to the pleasantly sour taste, while the formed acetic acid produces an acidic and astringent off-flavour (*114*).

## EXOPOLYSACCHARIDES PRODUCED BY AAB

Microbial polysaccharides are produced by a wide range of bacteria, presenting extreme diversity in terms of chemical structure and composition (*115*). AAB, for example, can produce large amounts of EPS, including both homopolysaccharides such as levan and bacterial cellulose and heteropolysaccharides such as acetan or xylinan and gluconacetan (*116*–*118*). Due to the importance of levan and bacterial cellulose (Fig. 1h) in research and industrial applications, some of their characteristics and main applications in the food industry are discussed in this paper.

### Levan

**Fig. 2:**
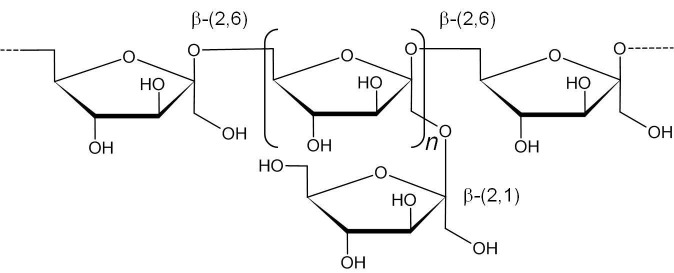
Chemical structure of levan

Levan is a polymer composed of d-fructofuranosyl residues joined by β-(2,6) bonds in the main chain and β-(2,1) bonds in the side chain, in addition to having a d-glucopyranosyl residue at the end of the main chain (*119*). Fig. 2 shows the structure of the levan. Levan is commonly biosynthesized by a restricted number of plants in the nature, but it can also be produced by several microorganisms, including Archaea, fungi and a wide range of bacteria (*58*, *120*). Among the AAB, species of the genera *Acetobacter, Gluconobacter, Gluconacetobacter, Kozakia, Komagataeibacter, Tanticharoenia* and *Neoasaia* are also capable of producing it (*54*–*58*) (Table 1).

Levan is synthesized and polimerized in the extracellular matrix by the action of the enzyme levansucrase, whose main function is to transfer fructose residues from sucrose by transfructosylation reactions (*121*). The enzyme has high specificity for sucrose and lower activity for fructose, mannose, raffinose, mannitol, *etc.* On the other hand, inhibition is observed in the presence of glucose and other sugars that have a configuration similar to glucose, such as lactose, galactose and maltose, as well as other sugar alcohols (*121*, *122*).

In general, the structure of levans produced by different organisms is similar but differs in terms of molecular mass, degrees of polymerization (DP) and branching (*122*, *123*). Plant levans have a lower molecular mass and DP than bacterial levans. Plant levans have a molecular mass of 2000 to 33000 Da and a DP<100, whereas bacterial levans have multiple branches (2 to 12%) and a molecular mass of 2 to 100 million Da with DP>100 (*121*, *123*, *124*).

In comparison with polysaccharides formed by pyranose rings, the structural characteristic of levan, in the form of furanose rings, plays an important role in the conformation of molecules in a solution, providing additional flexibility. Furthermore, the semiflexible chain of the rings interacts intramolecularly and intermolecularly, resulting in a densely packed spherical structure and aqueous solutions of low viscosity (room temperature) at concentrations where other polysaccharides would form pastes or gels (*121*, *122*, *124*–*126*).

Xu *et al.* (*127*) observed that aqueous solutions of levan (*Brenneria* sp. EniD312) exhibit Newtonian fluid behaviour at low content (3%; *m*/*V*) and non-Newtonian fluid (pseudoplastic fluid) behaviour at high contents (6, 9 and 12%; *m*/*V*) when studying the rheological properties of levan. Levan solutions derived from *Zymomonas mobilis* and *Erwinia herbicola* showed similar results (*128*). At amounts between 1 and 8%, however, the behaviour of *Bacillus subtilis* levan solutions was completely Newtonian (*128*). According to Xu *et al.* (*127*), levan could be a good additive in the food industry, since its non-Newtonian behaviour is interesting for the manufacture of dairy products, syrups and salad dressings.

Levan solutions also exhibit an atypical behaviour when compared to other polysaccharides, in which gel formation is not observed (*129*, *130*). However, Jakob *et al.* (*131*), when establishing the structure/function relationship of isolated AAB levans, suggested that in a solution, increasing their molecular mass reinforces intramolecular interactions to achieve a more compact structure characteristic of a ’microgel’ with hydrocolloid properties. The authors also emphasise that levans produced by AAB may thus offer new possibilities for applications in food.

Unlike many other polymers, levan does not swell in water, but it has a high solubility in hot water, a variable solubility in cold water and is insoluble in most organic solvents, with the exception of dimethyl sulfoxide (DMSO). The high solubility of levan in water is mainly attributed to its β-(2,6) bond rather than to the β-(2,1) bond, and the ramifications could only be a supporting factor (*121*, *122*, *126*). Levans are nonreducing agents that are not hydrolyzed by yeast invertases or amylases, but they are susceptible to acid hydrolysis (*122*). They decompose at approx. 225 °C, and their glass transition temperature is 141 °C (*126*). Another important property of levan is its adhesive strength. Although sugars are characterized by stickiness, the adhesive strength of levan is significantly higher than that of other natural polymers. The branches contribute to its cohesive strength, and the ability to form adhesive bonds with a wide range of substrates is given to its large number of hydroxyl groups. Levan is commonly referred to as a ’green’ adhesive because it is water-removable and has high-value applications in several areas (*8*, *121*, *125*).

**Fig. 3:**
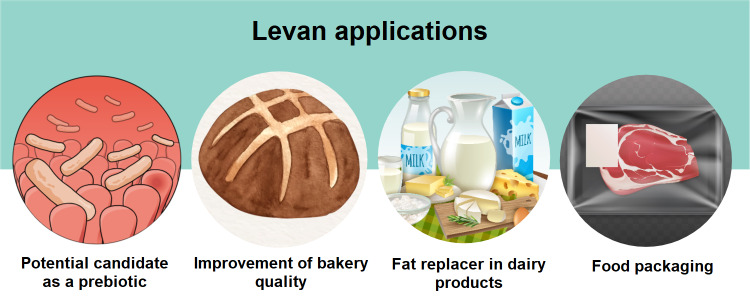
Levan applications in food area. Parts of the figure designed by Freepik

In the food area, several studies have explored the effects of levan-type fructooligosaccharides (L-FOS) and levan as prebiotics on probiotic bacteria and the complex gut microbiota; however, there is no conclusive evidence from human trials (*132*–*136*) (Fig. 3). In animal models, for instance, levan supplementation increased *Lactobacillus* and *Bifidobacteria* viability, while inhibiting *Escherichia coli* and *Clostridium perfringens* (*136*, *137*). Adamberg *et al.* (*132*), using the human faecal microbiota, reported that levan alters the composition of the faecal microbiota and the profile of metabolites, making it a potential candidate for prebiotics. Using metagenomic sequencing to assess the prebiotic activity of levan, Cheng *et al.* (*138*) also verified alterations in the intestinal microbiota of mice and the stimulation of the production of short-chain fatty acids.

In bakery, EPS are known to improve the rheological properties of dough and the texture, nutritional value, shelf life and machinability of wheat, rye and gluten-free bread (*56*). Jakob *et al.* (*139*) evaluated the functional effects of different AAB levans (*G. frateurii* TMW 2767, *G. cerinus* DSM 9533, *N. chiangmaiensis* NBRC 101099 and *K. baliensis* DSM 14400) added to wheat-based bread. The addition of *w*(EPS)=1 or 2% to the flour resulted in an increase in the volume, a noticeable softening of the fresh bread and a delay in the hardening of the bread after a week of storage. Although LAB and yeasts are common microorganisms in the sourdough (*54*), Hermann *et al.* (*56*) reported that AAB, such as *N. chiangmaiensis* NBRC 101099 and *K. baliensis* DSM 14400, can grow on a variety of flour types (wheat, whole wheat, spelled and rye) and produce large amounts of levan. Later, gluten-free bread types were made with buckwheat and molasses dough fermented by *G. albidus* TMW 2.1191 and *K. baliensis* NBRC 16680, and their volume, crumb hardness and sensory characteristics were evaluated (*54*). Bread made from the sourdough had better sensory and quality characteristics, such as higher specific volume and lower crumb hardness. However, the authors pointed out that strong acidification during fermentation could become a challenge in a large-scale production (*54*).

Other advantages of applying levan in foods include its use as a fat substitute. Fructans have fat-like properties that improve the flavour and spreadability of dairy products. Furthermore, high-molecular-mass levans are rarely detected by taste sensors, and odour detection is almost imperceptible due to their low volatility (*120*). Their low viscosity and high solubility make them an excellent substitute for gum arabic, as the latter also has excellent stabilizing and emulsifying properties for food applications (*122*). In the food packaging industry, levan as a component in edible starch films increases their barrier and mechanical properties in addition to being a cost-effective alternative (*119*). Gan *et al.* (*140*) also developed levan/pullulan/chitosan edible films enriched with ε-polylysine and applied them to strawberry. As a result, they demonstrated that films could help preserve the postharvest strawberry quality by minimizing water loss, inhibiting microbial development and decreasing the respiration rate during storage.

Although this study is not focused on biomedical or cosmetic applications of levan, this EPS has shown several bioactive properties, including antitumour, antimicrobial, anti-inflammatory, hypocholesterolaemic, antidiabetic and immunostimulating activities (*120*, *121*). As a result, in addition to being a useful EPS for food production, its consumption alone or in food can provide several health benefits to the host.

### Bacterial cellulose

**Fig. 4:**
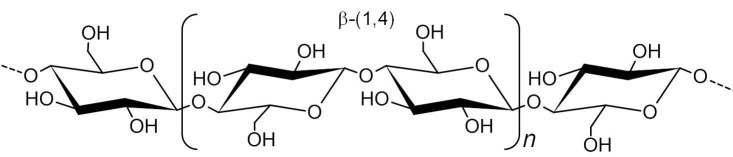
Chemical structure of bacterial cellulose

Bacterial cellulose is a linear glucan composed of several glucose monomers linked by β-(1–4) bonds (*8*) (Fig. 4). This biopolymer can be synthesized by various microorganisms, such as algae and fungi, as well as by various bacteria, including *Achromobacter, Alcaligenes*, *Aerobacter, Agrobacterium, Azotobacter, Gluconacetobacter, Pseudomonas, Rhizobium, Sarcina, Dickeya* and *Rhodobacter* (*141*, *142*). Among these bacteria, *Komagataeibacter* species (AAB) (Table 1) (*24*, *59*, *60*) are frequently utilized in research and commercial production, and are employed as model strains because of their high productivity and ability to metabolize a wide range of carbon/nitrogen sources (*60*).

**Fig. 5:**
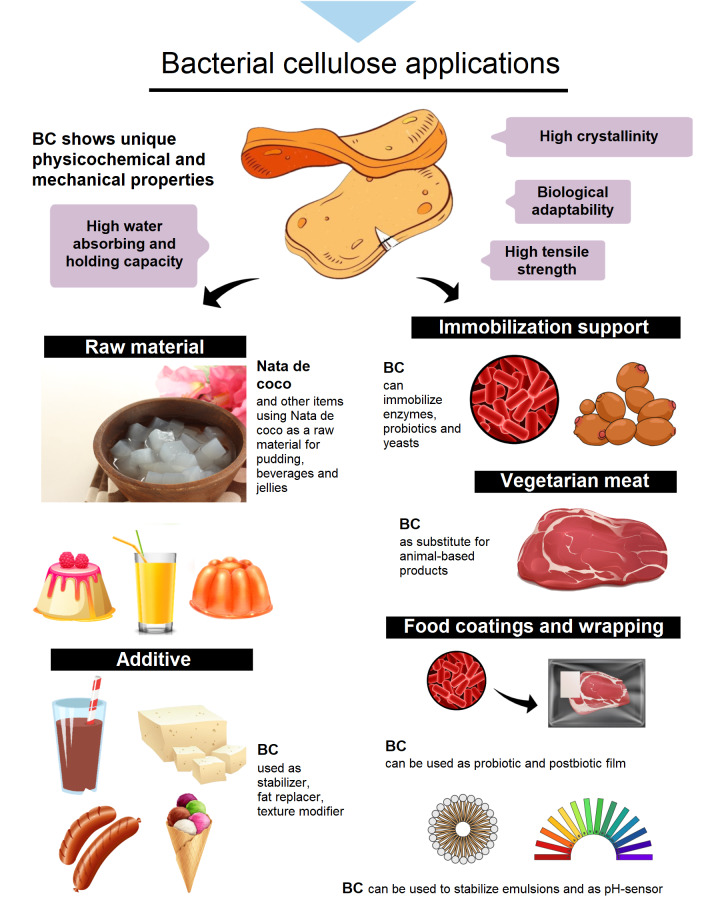
Different applications of bacterial cellulose (BC) in the food industry. Parts of the figure designed by Freepik and iStock

When cultivated under controlled conditions, *Komagataeibacter* produces highly porous bacterial cellulose structures in the form of pellicules (static culture) or as fibrous suspensions, pellets, spheres or irregular masses (agitated culture) (*142*, *143*). The synthesis of bacterial cellulose from glucose involves several individual enzymes, catalytic complexes and regulatory proteins. Briefly, β-glucan chains are formed first, followed by the assembly and crystallization of cellulose chains. In this final stage, the cellulose chains are released from the cell and self-assemble into fibrils (*144*). When compared to plant cellulose, bacterial celulose has a great number of unique physicochemical and mechanical properties, including higher crystallinity, degree of polymerization, water absorbing and holding capacity, tensile strength and biological adaptability (*60*). Moreover, plant-derived cellulose usually consists of hemicellulose and lignin, necessitating harsh chemical treatments to remove these impurities (*145*). Bacterial cellulose generated by microbial fermentation, on the other hand, has a greater purity and requires less energy and chemical processing for purification (*145*). As a result, bacterial cellulose has been used in a variety of food-related applications (Fig. 5) since it is a dietary fibre that has been approved as generally recognized as safe (GRAS) food by the US Food and Drug Administration (FDA) (*146*).

The ’nata de coco’, a traditional food consumed in the Philippines and other Southeast Asian countries, is one of the most well-known industrial applications of bacterial cellulose. In its manufacturing process, bacterial cellulose is synthesized by fermenting coconut water and then cleaning, washing, chopping and immersing it in sugar syrup to serve as a dessert (*142*, *145*, *147*). A variety of nata-like products have been developed to meet consumer demands. To change the colour and flavour of the dessert, different fruit juices, syrups and other ingredients have been employed. Other items containing nata de coco, such as fruit-flavoured pudding, drinks and jellies, have been marketed all over the world.

In other food systems, bacterial celulose has also shown promise as a stabilizer, noncaloric bulking agent and texture modifier. After heat sterilization, the use of an aqueous suspension of bacterial cellulose in liquids such as chocolate drinks prevents cocoa precipitation and stabilizes the dispersion (*148*). In pasty condiments, bacterial cellulose reduces the stickiness and controls the syneresis during storage (*148*). Furthermore, it promotes firmness in solid foods such as tofu, while it changes the texture of kamaboko by increasing hardness and fraturability (*148*). The addition of bacterial cellulose to meat products such as hamburger and sausage can also reduce fat content without compromising tenderness and juiciness, as well as produce stable emulsions (*148*).

Following the example of earlier products that were lower in calories, Guo *et al.* (*149*) demonstrated that adding bacterial cellulose/soy protein isolate blends to ice cream as a cream substitute can result in ice creams with low calories, melt resistance and good texture properties. Surimi products (*150*), cheese (*151*), meatballs (*152*), pork Frankfurters (*153*) and mayonnaise (*154*) are among other applications of bacterial cellulose as a fat replacer. These findings suggest that bacterial cellulose could be widely used as a food additive in processed foods to improve their quality and shelf life while also lowering the calories in the final products.

Bacterial cellulose has also been used as a vegetarian meat preparation when combined with *Monascus* extract obtained from a naturally red pigmented mould (*155*). The product has a natural meat flavour and is resistant to colour and morphological changes. Furthermore, because of its nonanimal origin, this ingredient may be a suitable substitute for animal-based products for some dietary restrictions (*60*, *142*).

It has attracted interest in research related to the immobilization of enzymes, cells and probiotics for use in food. Because of its superior characteristics compared to plant cellulose, bacterial cellulose provides stability to enzymes against temperature and pH variations (*156*). When compared to free laccase, laccase immobilized on magnetically modified bacterial cellulose showed superior thermal stability at 70 °C and maintained 65% of its initial activity after 8 cycles of use (*157*). Similarly, Chen *et al.* (*158*) obtained a retention of 69% of the original activity after seven recyclings when immobilizing fungal laccase on natural bacterial cellulose.

The bacterial cellulose has also been explored as a carrier for cell immobilization, primarily for yeasts in the winemaking process. This approach reduces inoculum preparation costs, as the yeast can be recovered and separated at the end of the fermentation process (*145*). BC has been shown to protect wine yeast from adverse conditions such as high osmotic pressure and low pH (*159*). As a result, the growth of immobilized yeast was better than that of free yeast (*159*). Furthermore, the metabolic activities of immobilized yeast in BC were reported to be higher than those of free yeast (*160*). Immobilized yeast in BC was also shown to have no negative impact on the sensory quality of the final product during repeated batch fermentation (*161*) and can increase the amount of alcohol produced compared to free cells (*162*).

Studies have proven that bacterial cellulose is an effective matrix for the immobilization of probiotic bacteria (*163*). In this context, Fijałkowski *et al.* (*164*) showed that as an immobilization support it improves probiotic viability, protecting against adverse conditions of the gastrointestinal tract (*164*). Furthermore, the authors established that the immobilization efficiency depends on the cellulose form, its synthesis and immobilization methods (*164*). Similarly, Phromthep and Leenanon (*165*) demonstrated that the bacterial cellulose produced from fruit juice residues and coconut milk improved the survival of immobilized *Lactobacillus plantarum* compared to free cells. Under prolonged incubation, Żywicka *et al.* (*166*) used the bacterial cellulose pellicle as a support for immobilization during prolonged incubation and reported that the cell viability of *L. delbrueckii* was affected after 72 h. On the other hand, the viability of *L. acidophilus* 016 immobilized in bacterial cellulose nanofibres was found to be 71% for up to 24 days when stored at ambient temperature (35 °C) (*163*). These findings show a potential because one of the requirements for a microorganism to be administered for therapeutic purposes is that it remains viable in the food that will be consumed (*164*).

More recently, bacterial cellulose has been reported as a matrix for probiotic films (*167*, *168*). The films can be used as coatings or wrapped over a variety of foods, providing consumer health benefits, as well as potentially inhibiting the growth of spoilage bacteria and fungi on food surfaces, thus extending the shelf life of the product (*167*, *168*). Similarly, the development of bacterial cellulose-based films and probiotic-derived bioactive metabolites (so-called postbiotics) has also gained attention for antimicrobial food packaging (*169*, *170*). For meat applications, the rapid release of postbiotics from the bacterial cellulose-based films into food is ideal for food with a finite shelf life, as it can effectively control foodborne pathogens such as *Listeria monocytogenes* while also extending the shelf life without affecting the sensorial attributes of the meat (*169*, *170*). In addition, several studies have performed *ex situ* and *in situ* modifications of bacterial cellulose to improve its properties for use in food packaging. However, to assess its potential usefulness as active packaging, more research is needed to investigate the mechanical properties, permeability, interactions and release rates in semisolid and solid food model media (*171*).

Other recent applications of bacterial cellulose include its use in dough leavening and baking trials to improve the rheological and sensory properties of gluten-free bakery products (*59*) and as a food-grade emulsion stabilizer (*172*–*174*), whose function can be extended to cosmetics and medical emulsions. In addition, bacterial cellulose can serve as an excellent matrix for the development of pH-sensitive indicators containing anthocyanins from different fruits, vegetables and flowers. Anthocyanins can display different colour spectra under acidic and basic conditions. Since the colour spectrum of each indicator has a direct link to pH changes in the food product, these pH-sensitive indicators can be used to track pH changes and monitor the freshness and spoilage of foods (fish, fruits and shrimp) and beverages (*175*-*179*).

## CONCLUSIONS

Acetic acid bacteria (AAB) are well known for causing wine spoilage. However, their importance and functionality in food applications were demonstrated in this review. Through oxidative fermentation, AAB can produce a variety of metabolites. Their role in various biochemical processes during food fermentation, such as vinegar, kombucha, water kefir, cocoa and lambic, results in unique sensory and biochemical characteristics, as well as health benefits. Although the commercial production of vinegar-based drinks is well established, research on fruit drinks fermented by AAB is still relatively new and scarce. Beverage production from fruits through gluconic acid fermentation is very promising since several fruits can be used (including nonstandard fruits, for example) and can be more adaptable to AAB metabolism than to the growth of LAB. Furthermore, due to the formation of gluconic acid and the presence of phenolic compounds in the fruits, the functional drink could meet the demand of lactose-intolerant consumers, those allergic to milk proteins, and those seeking a vegetarian, vegan and healthy diet. Due to its bioactive properties, levan could also be explored in beverage development. Levan could be produced *in situ* during the gluconic acid fermentation of fruit juices since several species of *Gluconobacter* can produce levan from sucrose and oxidize glucose to gluconic acid. Bacterial cellulose has numerous applications in food, with several products already marketed worldwide. However, similar to levan, its main challenge lies in the large-scale production and in reducing production costs to expand its applications in this area.
